# Mammalian Melatonin Agonist Pharmaceuticals Stimulate Rhomboid Proteins in Plants

**DOI:** 10.3390/biom12070882

**Published:** 2022-06-24

**Authors:** Lauren A. E. Erland, Christopher R. Dumigan, Jillian A. Forsyth, Liubov Frolova, Adam B. Yasunaga, Winnie Pun, Isaac T. S. Li, Michael K. Deyholos, Susan J. Murch

**Affiliations:** 1 Department of Chemistry, University of British Columbia, Kelowna, BC V1V 1V7, Canada; lauren.erland@ubc.ca (L.A.E.E.); jill.forsyth@hotmail.com (J.A.F.); liubovf@mail.ubc.ca (L.F.); adam.yasunaga@ubc.ca (A.B.Y.); winnie.pun@ubc.ca (W.P.); isaac.li@ubc.ca (I.T.S.L.); 2 Department of Agriculture, University of the Fraser Valley, Chilliwack, BC V6T 1Z4, Canada; 3 Department of Biology, University of British Columbia, Kelowna, BC V1V 1V7, Canada; christopher.dumigan@ubc.ca (C.R.D.); michael.deyholos@ubc.ca (M.K.D.)

**Keywords:** quantum dot, melatonin, serotonin, ramelteon, tasimelteon, rhomboid, receptor, rhomboid-like protein 7, gravitropism, morphogenesis

## Abstract

Melatonin is a human neurotransmitter and plant signalling metabolite that perceives and directs plant metabolism. The mechanisms of melatonin action in plants remain undefined. We hypothesized that roots have a melatonin-specific receptor and/or transporter that can respond to melatonin-mediating pharmaceuticals. To test this hypothesis *Arabidopsis* seedlings were grown with melatonin pharmaceutical receptor agonists: ramelteon and tasimelteon, and/or antagonists: luzindole and 4-P-PDOT. Ramelteon was found both to mimic and competitively inhibit melatonin metabolism in plants. Due to the higher selectivity of ramelteon for the MT1 receptor type in humans, a sequence homology search for MT1 in *Arabidopsis* identified the rhomboid-like protein 7 (RBL7). In physiological studies, *Arabidopsis rbl7* mutants were less responsive to ramelteon and melatonin. Quantum dot visualizations of the effects of ramelteon on melatonin binding to root cell membranes revealed a potential mechanism. We propose that RBL7 is a melatonin-interacting protein that directs root architecture and growth in a mechanism that is responsive to environmental factors.

## 1. Introduction

Melatonin (N-acetyl-5-methoxytryptamine; MEL) was first discovered in living plants in 1997 [1], and has since been established as an important phytohormone and anti-stress molecule in more than 236 plant species [2]. Melatonin has been reported to mediate root system architecture, inducing lateral and adventitious root growth, inhibiting primary root growth, and promoting a highly branched root system that has also been associated with improved performance under diverse stresses [3,4,5]. MEL also serves a more general antioxidant role in plants, both acting as a potent direct antioxidant and inducing other stress defence mechanisms, including antioxidant enzymes [6] and moderating stomatal closure [7]. Recently, the first phytomelatonin receptor, PMTR-1, has been proposed in *Arabidopsis thaliana* (L.) Heynh. stomata, for which a homolog has since been characterized in maize (*Zea mays* L.) [7,8]. MEL mediates diverse plant growth, developmental and survival processes throughout the plant life cycle [9,10]. The mechanisms underlying MEL action are becoming increasingly well-defined; however, relatively little is known about the transport, localization, and signalling mechanisms of MEL in plants [11]. MEL has previously been reported to mediate root morphogenesis in *Hypericum perforatum* (L.) [12,13]. Recently, we described the transport and localization of MEL in *H. perforatum* roots where MEL is transported in a specific manner moving laterally and is apparently being transported from the roots to aerial portions of the plant via apoplastic transport [11]. This localization pattern was disrupted by heat or cold stress, apparently to serve an antioxidant and protective function [11]. MEL has also been reported to be transported from the roots to aerial regions in response to abiotic stress tolerance in *Citrullus lanatus* (Thunb.) Matsum. & Nakai [14] and *Dracocephalum kotschyi* Boiss [15]. Although the effects of melatonin on plant growth and survival are well established, much is still yet to be discovered about MEL signalling dynamics in plants.

MEL receptors are well defined in humans and mammals, with specific inhibitors available, thanks to their clinical significance [16]. The primary mammalian MEL receptors are classified into two subtypes: MT-1 and MT-2 [17], although several other MEL receptors have been identified, including MT-3 (non-brain locations) and GPR50 (non-mammalian organisms) [18]. The methoxy and acetoamide side chains of the MEL molecule have been identified as determining the binding affinity and activity of the MEL molecule, and side-chain modifications that can increase affinity have been exploited to create diverse inhibitors [17]. Ramelteon (RAM) and tasimelteon (TAS) are two selective but non-specific pharmaceutical MEL receptor agonists whose binding affinities and locations have been well characterized in the mammalian system, but have not been examined in the plant system [19,20,21]. RAM has a higher affinity for MT-1 than MT-2, whereas TAS has a higher affinity for MT-2 [22]. Several MEL receptor antagonists are also available, including 4P-PDOT, which is selective for the MT-2 receptor subtype [23], and luzindole, which has a higher affinity for the MT-2 receptor subtype, similarly to TAS [24].

We hypothesized that plants possess a receptor similar in structure to mammalian MT-1 and MT-2 receptors and are responsive to pharmaceuticals that affect melatonin metabolism. We used *Arabidopsis* seedlings in axenic culture with RAM, TAS, 4P-PDOT, and luzindole to investigate MEL receptor activity. Our data indicate MT-1 receptor antagonist activity, leading us to propose a candidate MEL receptor in plants. We characterized responses of *Arabidopsis* mutant lines deficient in rhomboid-like protein 7 (RBL7) and report for the first time that RBL7 interacts with MEL as either a receptor or transporter. This interaction was found to be critical for determination of root growth and architecture.

## 2. Materials and Methods

### 2.1. Growth Experiments

*Arabidopsis* seeds (Col-0) were sterilized (15 min, 10% bleach, Clorox^®^, Oakland, CA, USA) and washed (3 x) with sterile distilled water before plating on germination media in Petri plates (60 × 15 mm Polystyrene disposable sterile Petri plates; Fisher Scientific, Pittsburgh, PA, USA) composed of half strength Murashige and Skoog [25] (MS) medium (Phytotechnology Labs, Shawnee Mission, KS, USA), 3% sucrose, with pH adjusted to 5.7, and solidified with 0.22% phytagel (Fisher Scientific). Media were autoclaved at 121 °C at 18 psi for 20 min. Preliminary experiments were performed to determine treatment levels of MEL (100 µM), agonists (10 µM): luzindole (N-aceetyl-2-benzyltryptamine) and 4P-PDOT ((N-(4-phenyl-1,2,3,4-tetrahydronaphthalen-2-yl)propenamide)) and antagonists (10 µM): RAM (N-{2-[(8S)-1H,2H,6H,7H,8H-indeno [5,4,-b]furan-8-yl]ethyl}propenamide, TAK-375, Ramelteon, Rozerem^®^) and TAS ((1R, 2R)-N-[2-(2,3-dihydrobenzofuran-4-yl)cyclopropylmethyl]propanamide, Tasimelteon, Hetlioz^®^). Concentrations of melatonin were chosen based on previously reported biologically active concentrations of melatonin in modulation of root architecture in *Arabidopsis* [3,5]. Concentrations of antagonists/agonists were selected based on preliminary studies and application rates of other mammalian indoleamine inhibitors found to be biologically active in *Hypericum perforatum* [26]. RAM, TAS, 4P-PDOT, and MEL were purchased from Millipore Sigma (Mississauga, ON, Canada), prepared as 10 mg/mL stock solutions in ethanol, and added to the autoclaved media after they had cooled to 60 °C [27]. All media were prepared just prior culturing. Ten seeds were started per plate, with four replicate plates per treatment. To examine the potential interactions between MEL, RAM, and TAS treatment groups were MSO (negative control), MEL (100 µM, positive control), RAM (10 µM), TAS (10 µM) RAM (10 µM) + MEL (100 µM), TAS (10 µM) + MEL (100 µM), or RAM (10 µM) + TAS (10 µM). Ten seeds were started per plate, with eight replicate plates per treatment group.

After 10 days, growth data including fresh weight, hypocotyl length, number of roots, primary root length, and number of secondary roots were collected and collated for statistical analyses. After collection of growth data (10 days) explants were rinsed with sterile distilled water, patted dry, pooled by plate, and stored at −80 °C for chemical analysis.

### 2.2. Growth Conditions

Unless otherwise stated, cultures were maintained at 24 °C under cool white fluorescent lights (~40 µmole m^−2^ s^−1^, Philips, Somerset, NJ, USA) with a 16 h photoperiod.

### 2.3. Gravitropism Assays

Gravitropism assays were performed on 14-day old Col-0 seedlings grown on MSO, or MSO + 50, 100, 250 µM MEL, or 10 µM RAM prepared in petri dishes as described above. Seedlings were rotated 90° and root angle from a 0° axis measured in ImageJ after 24 h. Ten seeds were started per plate, with five replicates per treatment. Results from replicate experiments were combined prior to plotting and analysis.

### 2.4. Quantum Dot (QD) Conjugation

QD were conjugated to MEL (QD-MEL), RAM, or TAS as previously described [11]. Briefly, commercial QD stock solutions (QD ITK ™ 545 carboxyl quantum dots, 8 mM; Thermo Fisher Scientific, Waltham, MA, USA) were diluted four times in 10 mM borate buffer (pH 7.4; Thermo Scientific Pierce, Mississauga, ON, Canada) then reacted with 8.1 mM MEL, RAM, or TAS (10 mg/mL stock in methanol; Millipore Sigma, Etobicoke, ON, USA) and 1.2 mM 1-ethyl-3-(3-dimethylaminopropyl)carbodiimide (EDC; 10 mg/mL stock in dH_2_O; Thermo Pierce Scientific, Waltham, MA, USA) for 2 h with gentle shaking at room temperature. This solution was then diluted two-fold with borate buffer (50 mM, pH 8.3) and concentrated ten-fold (Amicon^®^ Ultra-4 Centrifugal Filter Unit, 3 KDa; Millipore Sigma; 10 min, 3000 g). This was repeated for five buffer exchanges with 50 mM borate buffer (pH 8.3). Conjugated dots were then diluted to 1 µM in 50 mM borate buffer (pH 8.3), sealed, and stored at 4 °C until use.

### 2.5. QD Exposure

To determine effects of RAM and TAS on uptake and localization patterns in *Arabidopsis* roots, seven-day-old seedlings were removed from solid medium and moved to liquid MSO medium (3 mL, same as described less phytagel) in petri dishes (60 × 15 mm, Fisher Scientific, Hampton, NH, USA) containing 0.1 mM RAM (Millipore Sigma, St. Louis, MO, USA) or TAS (Millipore Sigma, St. Louis, MO, USA). Cultures were incubated with one seedling per plate, with three replicate plates per treatment, and allowed to incubate for 2 h to allow for uptake of inhibitor prior to addition of QD-Mel (2 nM), then allowed to incubate overnight (~16 h) prior to imaging. For localization of the agonists RAM and TAS themselves, QD-RAM or QD-TAS were added as described for QD-MEL without further addition.

### 2.6. QD Imaging and Processing

Imaging was performed using an inverted epifluorescence microscope (IX83, Olympus) with an automated stage (ASI) and focus maintaining mechanism, as previously described [11]. Image stitching was performed with CellSens (Olympus, Tokyo, Japan). Images were acquired using a 40x objective with an EMCCD camera (Andor iXon Ultra897). White LED illumination (X-Cite 120LED, Excelitas, Waltham, MA, USA) combined with mCherry fluorescence filters was utilized to visualize QD labelled MEL, RAM, and TAS. Unconjugated QD were previously shown not to be taken up by the plant roots [11,28,29], and this was confirmed with the current studies. Plant imaging was performed in a custom-made liquid chamber. All images were acquired at room temperature (23 °C). Images were processed using a combination of object identification using ilastik [30] and custom-written code in MATLAB. Both quantum dots and cell boundaries were identified by pixel classification via supervised machine learning using ilastik. All images were subjected to the same processing procedures. The centroid of each QD was identified and its proximity to a nearby cell boundary was determined by the probability map of cell boundaries. This allowed us to produce a histogram of the likelihood of QDs in proximity to cell boundaries. The fraction of QD localized to cell boundaries was determined by the total fraction of QDs above a probability threshold of 0.2.

### 2.7. Phytochemical Analysis

Samples were homogenized in 80:20 methanol (Optima Grade, Fisher Scientific, Mississauga, ON): 0.5 N tricholoracetic acid (TCA; Sigma, Mississauga, ON, USA) in 18 mΩ E-Pure water™ (Millipore, Burlington, MA, USA) with a disposable tissue grinder (Kontes Pellet Pestle; Fisher). Samples were centrifuged (13,000× *g*, 3 min), and supernatants were decanted and filtered (0.2 µm, Ultrafree-MC filtered centrifuge tubes; Millipore, Burlington, MA, USA) prior to chromatography. Serotonin (RT 0.77) and MEL (RT 2.49) were separated on a reverse-phase column (30 × 3 mm, 2.6 μm C18 100 Å, Phenomenex, Torrance, CA, USA) using an Acquity I-Class binary solvent manager (BSM) UPLC (ultra performance liquid chromatography; Waters, Mississauga, ON, USA) over a gradient of 0.1% formic acid (Eluent A) and acetonitrile (Eluent B) [(A%:B%)]: 0.0–0.5 min, 90:10; 0.5–3.5 min, 40:60; 3.5–4.2 min, 5:95; 4.2–6.5 min, 5:95; 6.5–7.0 min, 90:10], with a flow rate of 0.3 mL/min. Analytes were identified and quantified with a triple quadrupole tandem mass spectrometer (Xevo TQ-S, Waters) using previously optimized settings [31]: capillary voltage, 3500; desolvation gas rate, 800 L/hr; cone gas, 150 L/hr; desolvation temperature, 550 °C; source temperature, 150 °C, in multiple reaction monitoring (MRM) mode. For serotonin, transitions were 177 > 160 and 177 > 115, collision voltages of 10 V and 27 V, respectively, and cone voltage 45 V; for MEL, transitions were 233 > 159 and 233 > 174 with collision voltages of 23 V and 15 V, respectively, and cone voltage of 30 V. The limit of detection (LOD) was determined at approximately 10 pg on the column by the lowest concentration with an observed signal (>3 S/N) for all compounds. Quantification was done by comparison to authentic standards.

### 2.8. Sequence Alignment and Identification of RBL7 as a Candidate for a MEL Interacting Protein

Based on the results of growth experiments with the antagonists and agonists, we hypothesized that the target of MEL and RAM would have a higher homology to the human MT1 receptor type than to MT2 receptor type, as luzindole, 4-P-PDOT, and TAS, which all have higher affinity to the MT2 receptor subtype, showed reduced activity. In contrast, RAM, which has higher relative affinity for the MT1 human MEL receptor, showed the capacity to mimic MEL treatment in *Arabidopsis* seedlings. We therefore performed a BLASTx protein search for protein candidates in *Arabidopsis* with sequence similarity to the human MT1 (NP 005949.1) receptor. The predicted protein structure of RBL7 was acquired from the AlphaFold Protein Structure Database for Uniport accession O82756 under a CC-BY-4.0 license and is shown in Supplementary Figure S1 [32,33,34].

### 2.9. Response of RBL7 Mutants to RAM or MEL Treatment

To examine the potential role of RBL7 in responses to MEL, we examined the effect of MEL and RAM treatment on several *rbl7* mutant Col-0 background *Arabidopsis thaliana* lines. The *rbl7* mutant lines were obtained from the *Arabidopsis* Biological Resource Center (ABRC; CS717300-307). Seeds were sterilized and plated as described for Col-0 lines above, with Col-0 included as a control. Treatments were: MSO, 100 µM MEL, 10 µM TAS, or 10 µM RAM. Four replicate plates with 8 seeds each were started per treatment. Growth data were collected as described above. Prior to plotting, data across all mutants (CS707300-307) were averaged.

### 2.10. Confirmation of Insert in rbl7 Mutant Lines

*Arabidopsis* accessions were tested for the presence of a T-DNA insertion mutation in the RBL7 gene via PCR and gel electrophoresis according to the GABI-Kat confirmation strategy [35]. Primer sequences were obtained for the RBL7 T-DNA insertion sequence from the GABI-Kat website. The WT reverse primer was designed by downloading the AT4G23070 genomic sequence from Phytozome, with additional 200 bp upstream and downstream, and finding the insertion flanking sequence from the GABI-Kat website. *Arabidopsis* accession DNA was extracted using EZ-10 Spin Column Plant Genomic DNA Miniprep Kit (BioBasic, Markham, ON, Canada) according to manufacturer′s instructions. PCR was conducted using DreamTaq PCR Master Mix (2X) (Thermo Scientific, Waltham, MA, USA) with primer sets HN48 + 8409 for detection of T-DNA insertion at gene specific locus and HN48 + 479 for detection of WT allele (Table 1).

PCR conditions for amplification were: 94 °C for 2 min, 37 cycles of 94 °C for 30 s, 59 °C for 30 s, 72 °C for 90 s, followed by 72 °C for 5 min. Amplicons were then run on a 1% agarose gel in TAE buffer at 100 V for 45 min to visualize the presence of T-DNA insertion and WT alleles.

A chi square test was performed in Prism (v9.3.1, GraphPad LLC, San Diego, CA, USA) to determine if the population distribution varied from an expected 1:2:1 (WT: heterozygous: homozygous) ratio.

### 2.11. Statistical Analysis

All statistical analyses were performed in Prism, and the significance level was set to alpha = 0.05. All experiments were repeated at least twice. Individual seeds were treated as pseudoreplicates and averaged by plate. Growth data in Col-0 were found to be normally distributed and were analysed by one-way ANOVA with the Holm–Sidak multiple comparison model. Growth data for mutant lines were analysed by two-way ANOVA with the Sidak multiple comparison model between Col-0 (wild-type) and RBL7 for each treatment. Gravitropism data were analysed using ANOVA with a linear trend (linear contrast) multiple comparison model in Prism.

## 3. Results

**Figure 1:**
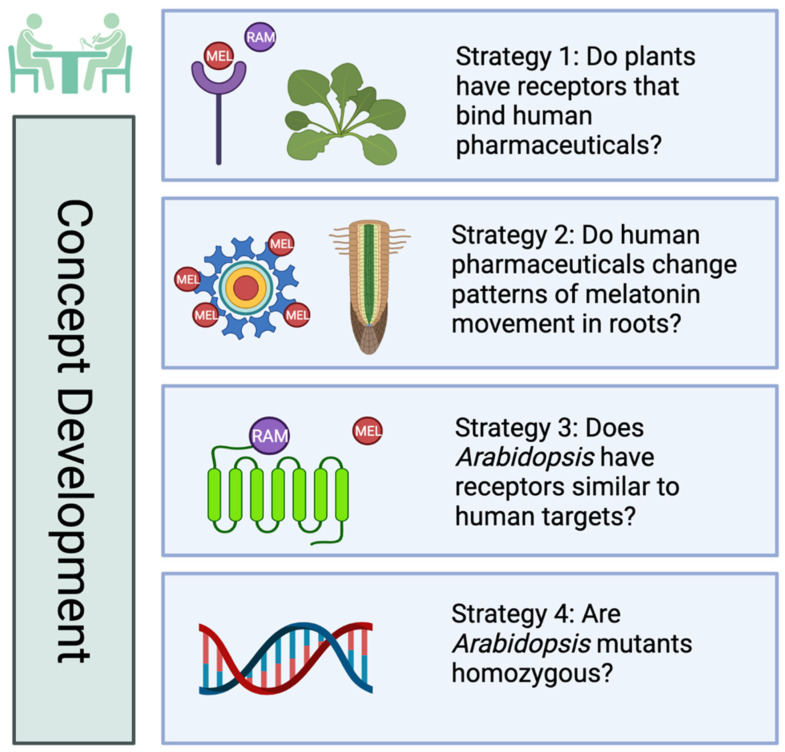
Overview scheme of the experiments included in the study. This image was created in www.biorender.io (accessed on 22 June 2022).

An overview scheme of the experiments performed is provided in Figure 1.

### 3.1. Effects of MEL, Agonist and Antagonist Treatment on Growth and Regeneration in Col-0

**Figure 2:**
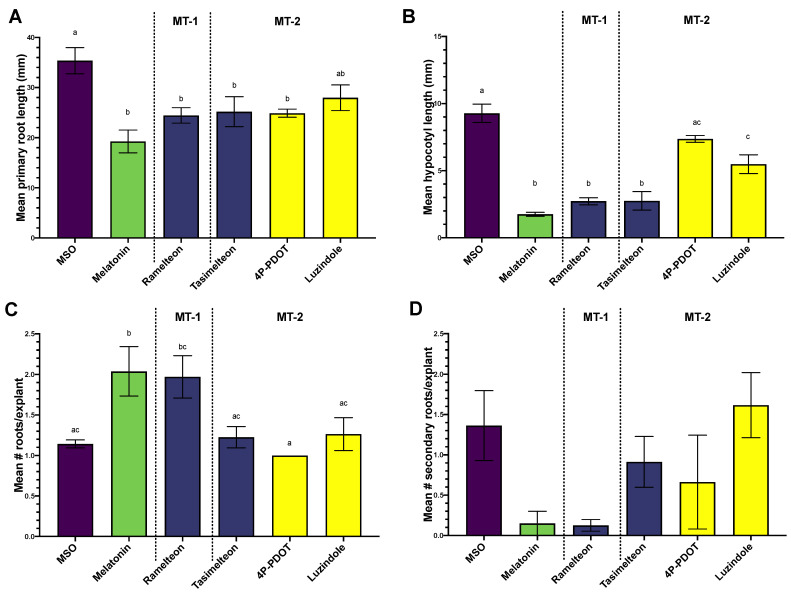
Growth effects of treatment with melatonin (100 µM) and melatonin agonists ramelteon (10 µM) and tasimelteon (10 µM) and antagonists luzindole (10 µM) and 4P-PDOT (10 µM) in *Arabidopsis thaliana* Col-0 (**A**) mean primary root length, (**B**) mean hypocotyl length, (**C**) mean number of primary roots/explants, (**D**) mean number of secondary roots/explants. Data are displayed as mean; error bars extend to the range of standard error. Different letters indicate significant difference between groups by ANOVA with Tukey’s honestly significant difference multiple comparisons model with alpha = 0.05. Where not shown, there was no significant difference between groups. MT-1 indicates compounds with higher affinity for the human MT-1 receptor subtype; MT-2 indicates compounds with higher affinity for the human MT-2 receptor subtype. Agonists have bars shaded in blue, whereas antagonists have yellow shaded bars.

Treatment of *Arabidopsis* seedlings with MEL increased the number of roots (Figure 2A), while decreasing the number of secondary roots (Figure 2D), root (Figure 2C), and hypocotyl length (Figure 2B). Treatment with RAM, the agonist with higher affinity for the MT-1 receptor subtype, was not significantly different from treatment with MEL across all growth parameters measured (Figure 2). Growth responses to TAS or MT-2 selective antagonists were more variable. TAS showed a comparable response to MEL and RAM in reducing primary root (Figure 2A) and hypocotyl length (Figure 2B), but was not significantly different from control in induction of primary (Figure 2C) or secondary roots (Figure 2D). Antagonists generally had intermediate and non-significant effects on all growth parameters, with two exceptions: luzindole mediated reduction in hypocotyl length was significantly lower than control (MSO) treatment, but significantly higher than MEL treatment (Figure 2B), and 4P-PDOT mediated reduction in primary root length was significantly reduced compared to the control, but not significantly different from MEL (Figure 2C).

**Figure 3:**
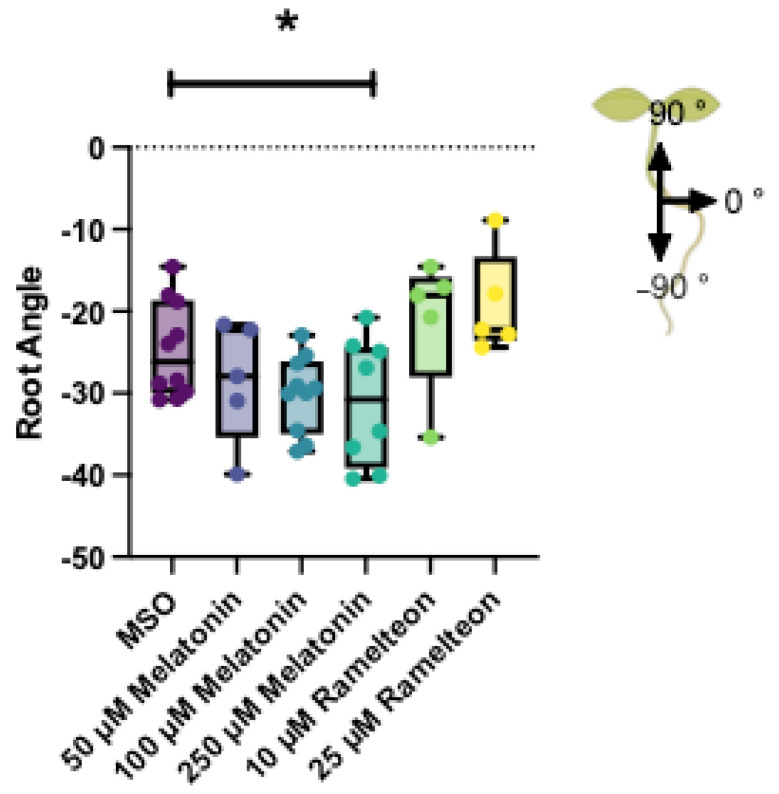
Gravitropic response of 14-day old *Arabidopsis thaliana* Col-0 seedlings in response to melatonin (blues) or ramelteon (green/yellow) treatment. Whiskers extend to the maximum and minimum values; boxes extend to first and third quartiles and central bar mean. Star indicates a significant linear trend (linear contrast) with increasing melatonin concentration using ANOVA with multiple comparisons, α = 0.05.

Whereas MEL showed a dose dependent inhibition of the gravitropic response (Figure 3), RAM did not have an effect on the gravitropic response.

### 3.2. Phytochemical Quantification

Consistent with previous studies, MEL concentrations in untreated *Arabidopsis* seedlings were <0.01 ng/g (Supplementary Figure S2). MEL supplementation of the media increased tissue concentrations, whereas supplementation with RAM and TAS did not significantly change the MEL concentration in the tissues (Supplementary Figure S2).

### 3.3. Localization

**Figure 4:**
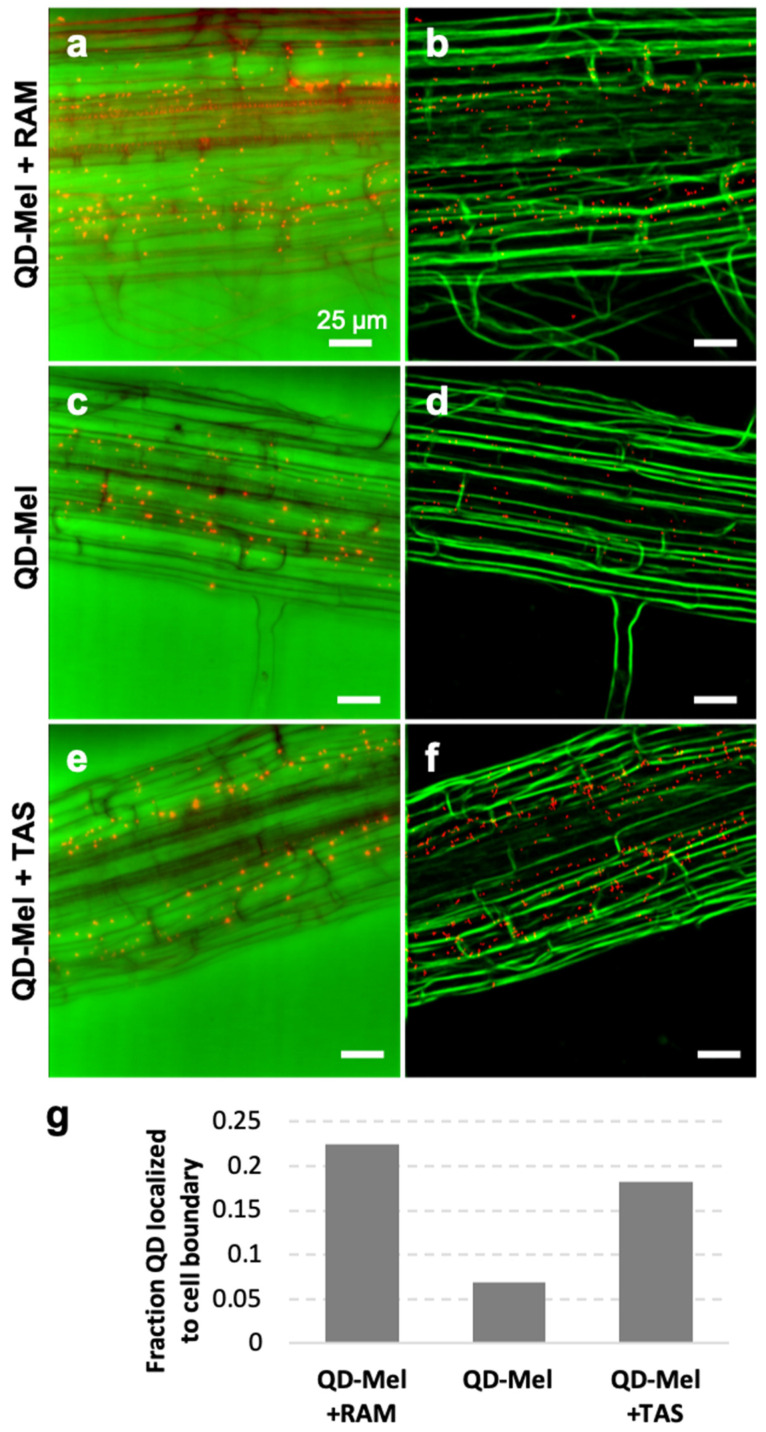
*Arabidopsis thaliana* roots treated with ramelteon + QD-MEL (**a**,**b**), QD-MEL alone (**c**,**d**), and tasimelteon + QD-MEL (**e**,**f**) seen under 40× magnification with mCherry fluorescent filter. Images are pseudocoloured to show QD-MEL in red and overlaid on brightfield images in green; (**a**,**c**,**e**) are 2D projections of raw Z-stacks; (**b**,**d**,**f**) are post-processed images where detected QD-MEL (red) are overlaid with the probability map of cell boundaries (green). All scale bars are 25 μm. Arrows in (**a**) and (**e**) show QD-MEL aligned with cell boundaries; arrow in (**d**) indicates QD-MEL clearly inside cells. (**g**) Quantification of QD-MEL localization to cell boundaries, showing that QD-MEL + RAM and +TAS are significantly more localized to cell boundaries than QD-MEL alone.

RAM and TAS modified uptake patterns of QD-MEL in *Arabidopsis* roots (Figure 4). Treatment with agonists did not prevent uptake of QD-MEL (Figure 3); instead, it appeared to block entry of the QD-MEL into some cell types. QD-MEL appeared to remain in the apoplastic space and did not move once blocked. Machine learning algorithms correlating QD-MEL distances to cell boundaries indicate that this effect was greater with RAM (Figure 4a,b) than with TAS (Figure 4e,f). QD-MEL treatment alone shows poor correlation with cell boundaries (Figure 4c,d). The fraction of QD colocalized to the cell boundary was quantified in Figure 4g, showing significantly higher fraction of QD-MEL with either RAM or TAS localized to cell boundaries compared to QD-MEL alone.

### 3.4. Identification of RBL7 as a Candidate for a MEL Interacting Protein

A BLASTx search of the full sequence of the MT1A mammalian melatonin receptor identified a single candidate match: RHOMBOID-like protein 7 (AtRBL7; NP 194028.1) with a length of 313 amino acids. The match quality was low and statistically not significant: Score 29.6, Expect 8.5, Identities 14/35 (40%), Positives, 19/35 (40%), and Gaps 5/35 (14%).

### 3.5. Effects of MEL and Agonist Treatment in rbl7 Mutants

**Figure 5:**
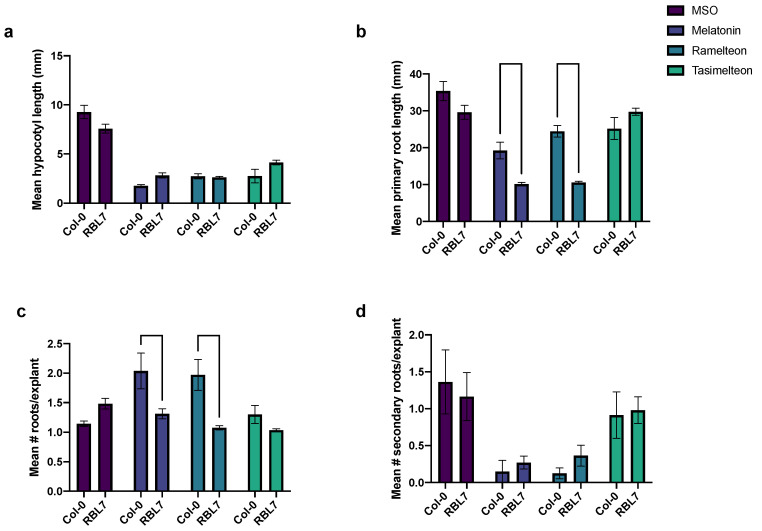
Growth effects (**a**) Mean hypocotyl length, (**b**) Mean primary root length, (**c**) Mean number of roots per explants and (**d**) Mean number of secondary roots per explant, of treatment with melatonin (100 µM) and melatonin agonists ramelteon (10 µM) and tasimelteon (10 µM) in *Arabidopsis thaliana* Col-0 and rhomboid-like protein 7 (RBL7) mutant seedlings. Data are displayed as mean, error bars extend to the range of standard error, bars between treatments indicate significant differences by analysis of variance with Sidak multiple comparison model between Col-0 and *rbl7* mutant (alpha = 0.05) between WT and *rbl7* mutant under a particular treatment, where not indicated, no significant difference was observed.

From the *Arabidopsis* lines CS717300-307, three were wild type and five were heterozygotes with the T-DNA insertion; no homozygotes were identified (3:5:0, *p* < 0.05, chi square 34.1). When the heterozygote line 302 was self-crossed, a ratio of 8:2:0 was seen, which differed significantly from an expected 1:2:1 ratio (*p* < 0.05, chi square 16.4). No significant phenotypic differences in hypocotyl length (Figure 5a), primary root length (Figure 5b), number of roots (Figure 5c), or number of secondary roots (Figure 5d) were observed between the wild-type and *rbl7* mutant grown on MSO or TAS (Figure 5). Treatment with MEL, TAS, or RAM led to a significant reduction in hypocotyl length, primary root length, and number of secondary roots, regardless of genotype (Figure 5a,b,d, respectively), consistent with other results in this study. In the Col-0 control, treatment with MEL or RAM increased the mean number of roots per explant, with the number of roots being significantly lower in the RBL7 than Col-0 for each treatment and equal to MSO untreated levels (Figure 5c). The reduction in primary root length inhibition was exaggerated with RAM and MEL treatment in the *rbl7* mutant as compared to the control, with the *rbl7* mutants having significantly shorter root lengths as compared to Col-0 in these treatments (Figure 5c).

## 4. Discussion

MEL is a relatively novel and important phytohormone that is important in the physiology and development of plants and particularly in root system architecture, as well as being a potent antioxidant enabling better plant performance under both biotic and abiotic stress [3,6,9]. Melatonin is known to interact with diverse plant signalling networking including phytohormone networks and plant signalling cascades including calcium/calmodulin and mitogen-activated kinases [12,15,36,37]. Understanding signalling mechanisms of MEL in plants has enormous implications for our understanding of plant responses to their environments. The search for MEL interacting proteins in plants has led to one MEL receptor, PMTR1, which, to date, has been identified in *Arabidopsis* and maize [7,8], although some concerns exist as to the whether the protein represents a bona fide receptor or whether it is a melatonin interacting protein [38]. Several downstream mediators have been proposed [39]; however, the initial steps in the plant MEL signalling cascade remain elusive. An approach that has been considered in the past is the use of mammalian indoleamine inhibitors to identify potential homologies to mammalian MEL interacting proteins in plants; however, this is the first report of the application of MEL receptor agonists in plants.

Our growth experiments showed that treatment with RAM, an agonist with a strong specificity for the MT1 receptor subtype, resembled MEL treatment, whereas imaging experiments with QD-MEL demonstrate that both RAM and TAS modified uptake patterns of MEL. This response appears to be specific to auxin-independent growth as well. MEL was able to inhibit the gravitropic response, a classically auxin mediated process; RAM was not able to mimic this effect [40]. MT1 and MT2 are seven transmembrane domain g-coupled receptors in mammals [18]. RAM has a higher specificity for MT1, whereas TAS as an agonist of both MT1 and MT2, has higher specificity for MT2 [20,21]. Based on these observations, we hypothesized that there is a phytomelatonin receptor with a similar structure to the mammalian MT1. A BLASTx search for proteins with homology to mammalian MT1 (NP 005949.1) in the *Arabidopsis* genome identified a seven-transmembrane domain RBL7 (NP 194038; At23070) with low sequence homology that is not functionally defined. The low homology was expected due to the difficulty in identifying phytomelatonin receptors and the low homology observed in the sole identified phytomelatonin receptor to date, PMTR1, which was reported to have only 9–15% sequence similarity with mammalian MEL receptors [7]. Interestingly, although in a different transmembrane domain, the candidate possesses several residues that are important for RAM binding in the human MT1 active site, including Ser110; Gly108, one of two ligand interacting residues in MT1, Val111 being the other ligand interacting residue; and Met107, which has been shown to be substituted by Thr107 in GPR50, an orphan MEL receptor [20,41]. The putative RBL protein possesses a TSGxV sequence, starting at residue 282 and the NRY motif downstream of this domain, which is found in both MT1 and MT2, although not in the PMTR1 phytoMEL receptor [7,19]. *Arabidopsis* mutants for RBL7 were found to have no significant phenotypic differences in the absence of indoleamine treatment, as has been previously reported. Previous authors have hypothesized that this is due to significant redundancy in the RBL system because of critical roles in development [42]. Treatment with MEL and RAM lead to a modification of response in the mutant lines preventing an increase in the number of roots seen in the wild type (a knockout of the expected response) and a significant decrease in primary root length (a hypersensitive response), implying that RBL7 may be important in MEL-mediated changes to root system architecture in *Arabidopsis*.

RBL proteins are a large and exceptionally diverse superfamily, originally identified as serine proteases; however, exceptions to this function are common [42]. More recent data indicate that many in this family do not function as proteases, and functions remain to be determined [43]. RBL7 is a mixed secretase-type rhomboid, predicted to localize to the plasma membrane, lacking intramembrane protease activity [43,44,45]. Rhomboid proteins have been described in the genomes of all species investigated and across every kingdom of life with described functions in signalling, development, apoptosis, and parasite invasion, with rhomboids increasingly becoming a target of interest in human disease due to their roles in mitochondrial function [43]. Rhomboids have been arranged into two main families: PARL (presenilins-associated rhomboid-like protein) and secretase type rhomboids. Secretases can be further broken down to include: secretase type A and B, and mixed secretases, which generally include most plant rhomboids and possess characteristics of both A and B. Rhomboid proteins are hypothesized to have evolved in early life forms, with the B secretase hypothesized to be the most ancient class [45]. Classically rhomboids either modify proteins for the activity or release signalling peptides or membrane bound transcription factors including NAC and bZIP [44,46] in plants, both of which have been reported to respond to MEL treatment and whose functions have been associated with stress responses [47]. These data may indicate that plants have retained, in some part, this highly conserved primary metabolic system through evolution.

**Figure 6:**
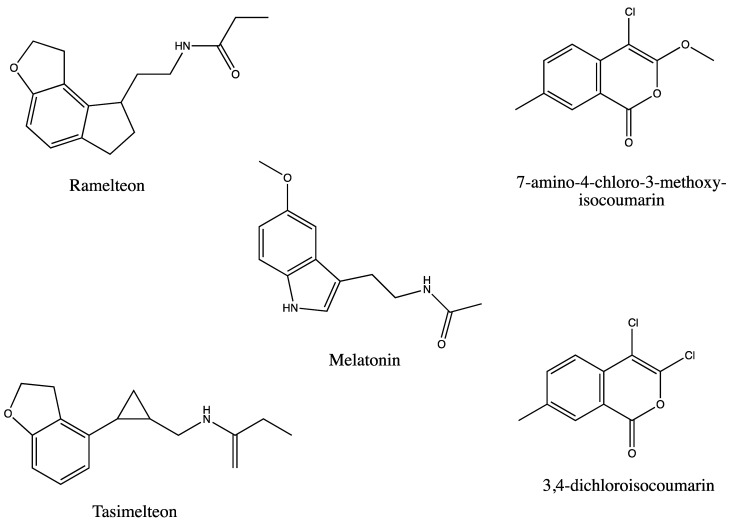
Structures of melatonin, mammalian receptor agonists ramelteon and tasimelteon, and rhomboid protein inhibitors 7-amino-4-chloro-3-methoxy-isocoumarin and 3,-dichloroisocoumarin.

Our data indicate that RAM and TAS are acting as competitive inhibitors of MEL, interacting with RBL7 in *Arabidopsis* roots either directly or through interactions with other components of a putative signalling pathway. Several rhomboid inhibitors have been reported, which show an interaction between the Ser-His catalytic dyad and carbonyl groups, suggesting that MEL or RAM may function to inhibit the activity of RBL7 (Figure 6). Interestingly, our results suggest that the *rbl7* mutation may be homozygous lethal as we saw a dramatic shift from expected population distribution of wild-type to heterozygous individuals with no homozygous individuals in our population of progeny resulting from the self-cross of one of the heterozygous lines. Although the competitive inhibition of RAM could be attributed to known competition between QD-labelled and unlabelled phytohormone at the binding site [28], the difference in disruption of localization between RAM and TAS and QD-MEL supports the fact that this is a more specific response. Evidence is available in the literature to support both an auxin-dependent and an auxin-independent mechanism for melatonin-mediated growth and development [3,4,48]. The differential response of seedlings to RAM and MEL in gravitropism assays suggests that RBL7 may also be associated with auxin-independent mechanisms of growth mediation by melatonin.

An interesting feature of rhomboids is their ability to move rapidly through biological membranes. This is due to their ability to disrupt lipid structure in the regions surrounding them and has been hypothesized to be responsible for some of their non-catalytic functions [49]. As we have noted in this and our previous studies, QD-MEL seemingly lines up at membranes before crossing [11], and visual observations seem to indicate an active uptake mechanism. Recent reports have hypothesized that proteolytically inactive rhomboids may disrupt membrane structure, leading to channelling of ligands towards receptors for transport [49]. If a rhomboid is interacting with MEL directly or with another MEL channel or transporter, it is possible that this interaction leads to channelling of MEL towards the opening, thereby enhancing MEL transport across the membrane. Further research is needed for a full understanding of the mechanism.

Interactions between the indoleamines MEL and serotonin and lipid metabolism also support a potential link between RBLs and MEL function. Recently, an investigation of the function of AtRBL10 found that it is involved in phosphatidic acid (PA) metabolism and lipid biosynthesis. The authors hypothesize that RBL10 functions to directly process components of a PA transport/synthesis complex responsible for the transport of PA between the chloroplast and the endoplasmic reticulum (ER) where synthesis occurs, or that it releases a signalling peptide that indirectly affects the process [50]. MEL treatment has previously been found to modify lipid metabolism, increasing PA levels as well as the downstream lipid metabolites diacylglycerol (DAG), phosphatidylinositol (PI), phosphatidylcholine (PC), and monogalactosyldiacylglycerol (MGDG) in sweet potato (*Ipomea batatas* L. Lam) [51]. In addition to potentially serving a function in membrane stabilization and stress responses, indoleamines have also been found to interact with PI turnover. Serotonin has been found to mimic the effects of red-light exposure in maize, inducing PI signalling turnover and downstream nitrate reductase activity and initiating calcium signalling cascades in maize, initiating phytochrome signalling [52,53]. More recent studies have also suggested that MEL could be involved in light signalling processes [54], particularly through further downstream interactions with the constitutive photomorphogenesis (COP) 9 signalosome, whose activation is currently poorly understood [55].

## 5. Conclusions

**Figure 7:**
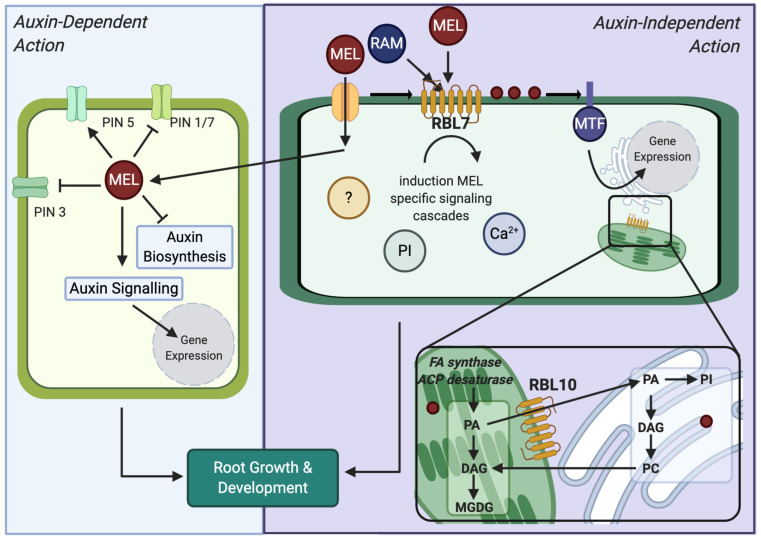
Proposed hypotheses for melatonin mediated root growth through auxin-dependent and independent actions. ACP, acyl carrier protein; FA, fatty acid; MEL, melatonin; MTF, membrane-bound transcription factor; PA, phosphatidic acid; PC, phosphatidylcholine; DAG, diacylglycerol; MGDG, monogalactosyldiacylglycerol; PI, phosphatidylinositol; RAM, ramelteon; RBL, rhomboid-like protein.

Our results demonstrate that the mammalian MT receptor agonist RAM is able to mimic the effects of MEL treatment on *Arabidopsis* Col-0 seedlings, reducing hypocotyl length, reducing primary root length, and increasing secondary root growth in a manner consistent with the effects of MEL treatment in *Arabidopsis*. Previous studies have understandably focused on potential interactions between MEL and the phytohormone auxin, which plays a major role in mediating root system architecture (among diverse and significant other functions) in plants [56]. There exist conflicting reports, however, in the literature as to whether the effects of MEL on root development is an auxin-dependent or -independent response [3]. Here, we propose that MEL has both auxin-dependent and auxin-independent effects on root development (Figure 7). We hypothesize that the auxin-independent signalling pathway is mediated by a mixed secretase-type rhomboid protein, possibly RBL7. The possibility of rhomboids being a MEL interacting protein is also supported by this ancient evolutionary presence, as MEL is hypothesized to have conveyed an evolutionary advantage to the first microorganisms that inhabited an oxygenating atmosphere [57].

The means by which MEL may interact with an RBL is unclear, but, based on a review of the literature, we hypothesize that it may take one of several forms (Figure 7). In brief, we propose the following hypotheses for rhomboid-mediated MEL action in plants:

**H1.**  *A proteolytically inactive RBL channels MEL towards a transporter or channel that transports MEL across the plasma membrane*.

**H2.**  *MEL interaction with a proteolytically active RBL leads to cleavage of membrane-bound transcription factors and activation of signalling cascades, e.g., COP9 signalosome, MAPK signalling, or calcium dependent kinases*.

**H3.**  *MEL interaction with an RBL similar to RBL10 induces trafficking of lipids between the ER and the chloroplast, leading to modifications in lipid biosynthesis that result in modifications in membrane compositions and/or induction of PI signalling*.

Further research is needed to investigate potential interactions between an *Arabidopsis* RBL and MEL. The potential for an interaction between this ancient molecule and an equally ancient receptor holds profound implications for our understanding of MEL signalling and function in plants and humans and has applications to both human health and improved crop security under changing global climate.

## Figures and Tables

**Table 1:** Primers used for TDNA insertion confirmation.

Primer Name	Description	Sequence
HN48F	Gene-Specific Primer	5′-ACAGTCCTAAAATCTCAAACCCAG-3′
8904R	TDNA Primer	5′-ATATTGACCATCATACTCATTGC-3′
479R	WT Primer	5′-GCACAATTCAACATGTTTCCA-3′

## Data Availability

Unprocessed image files included in the manuscript can be accessed through the Dryad database https://doi.org/10.5061/dryad.vx0k6djr5.

## Supplementary Materials

The following supporting information can be downloaded at: https://www.mdpi.com/article/10.3390/biom12070882/s1, Figure S1: Predicted protein structure of rhomboid-like protein 7 from the AlphaFold Protein Structure Database, Uniprot accession O82756. Figure S2: Melatonin content in *Arabidopsis thaliana* Col-0 tissues treated with 10 µM ramelteon, 10 µM tasimelteon, or 100 µM melatonin. Data are displayed as mean; error bars extend to the range of standard error. Figure S3: Gel images of GABI-Kat confirmation strategy for presence of T-DNA insertion in RBL7 gene in *Arabidopsis* lines used in this study. Figure S4: Gel images of GABI-Kat confirmation strategy for presence of T-DNA insertion in RBL7 gene in 10 progeny of self-crossed heterozygous *Arabidopsis thaliana* CS717302. Numbers 1–10 indicate individual progeny of the self-cross. Table S1: Summary of the GABI-Kat confirmation strategy and the genotype of the RBL7 gene for the *Arabidopsis thaliana* lines from ABRC (CS717300-307). Table S2: Summary of the GABI-Kat confirmation strategy and the genotype of the RBL7 gene for 10 progeny of self-crossed heterozygous *Arabidopsis thaliana* CS717302 Numbers 1–10 indicate individual progeny of the self-cross.
