# Supplementary material for: Mammalian Melatonin Agonist Pharmaceuticals Stimulate Rhomboid Proteins in Plants
Source: Biomolecules. 2022 Jun 24;12(7):882. doi: 10.3390/biom12070882 (PMC9313243; doi:10.3390/biom12070882)
Supplement: Supplementary file 1 [file biomolecules-12-00882-s001.zip › biomolecules-1779778-supplementary.pdf]

Supplementary Materials

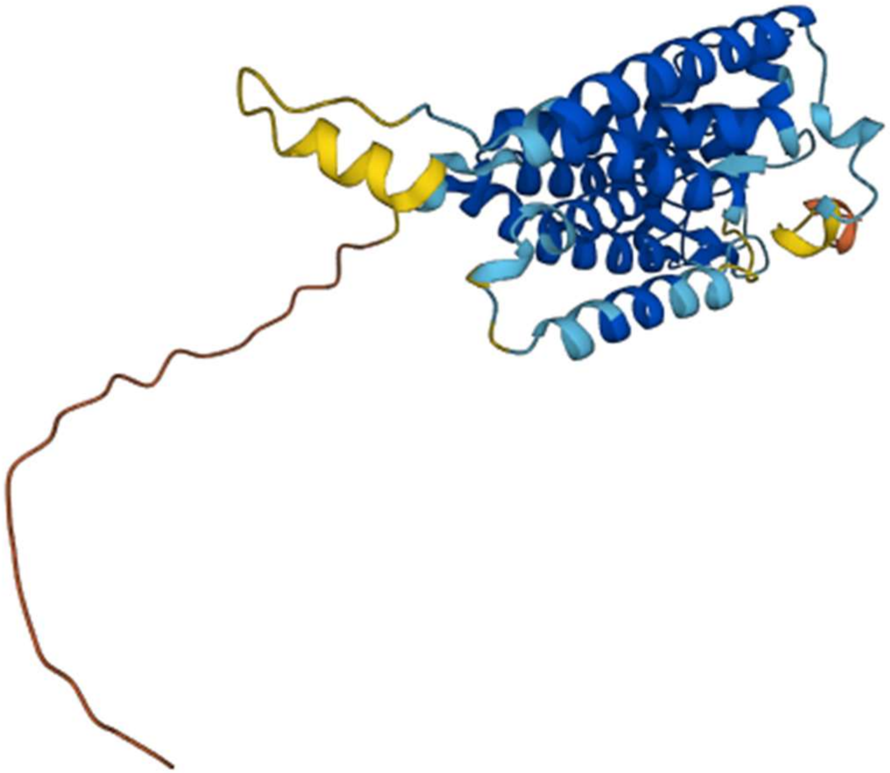

Figure S1: Predicted protein structure of rhomboid-like protein 7 from the AlphaFold Protein Structure Database, Uniprot accession O82756.

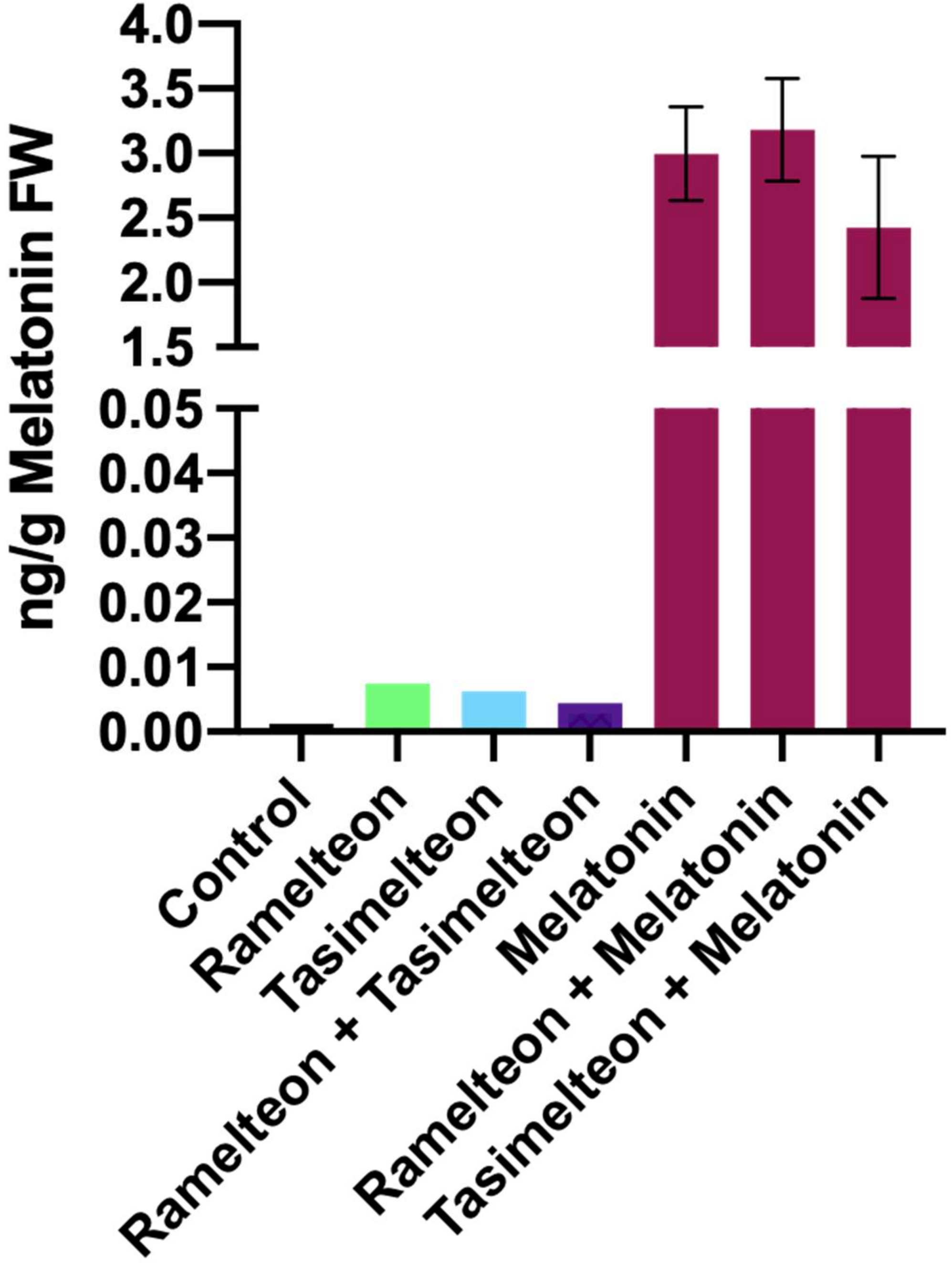

Figure S2: Melatonin content in Arabidopsis thaliana Col-0 tissues treated with 10 M ramelteon, 10 M tasimelteon, or 100 M melatonin. Data are displayed as mean; error bars extend to the range of standard error.

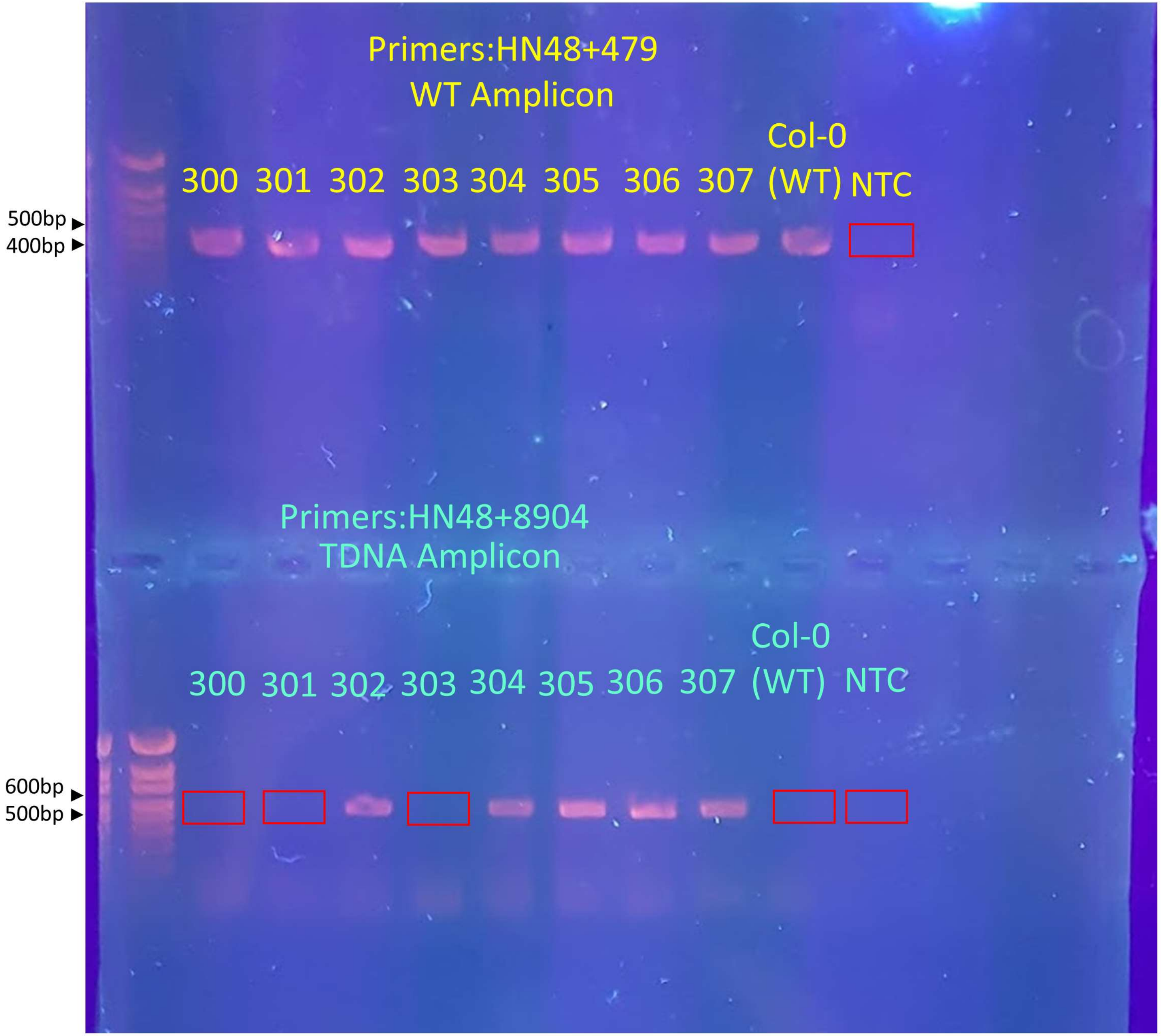

Figure S3: Gel images of GABI-Kat confirmation strategy for presence of T-DNA insertion in RBL7 gene in Arabidopsis lines used in this study

Primer Pair	Amplicon Size (bp)
WT (HN48+ 479)	479
TDNA (HN48 +8409)	504

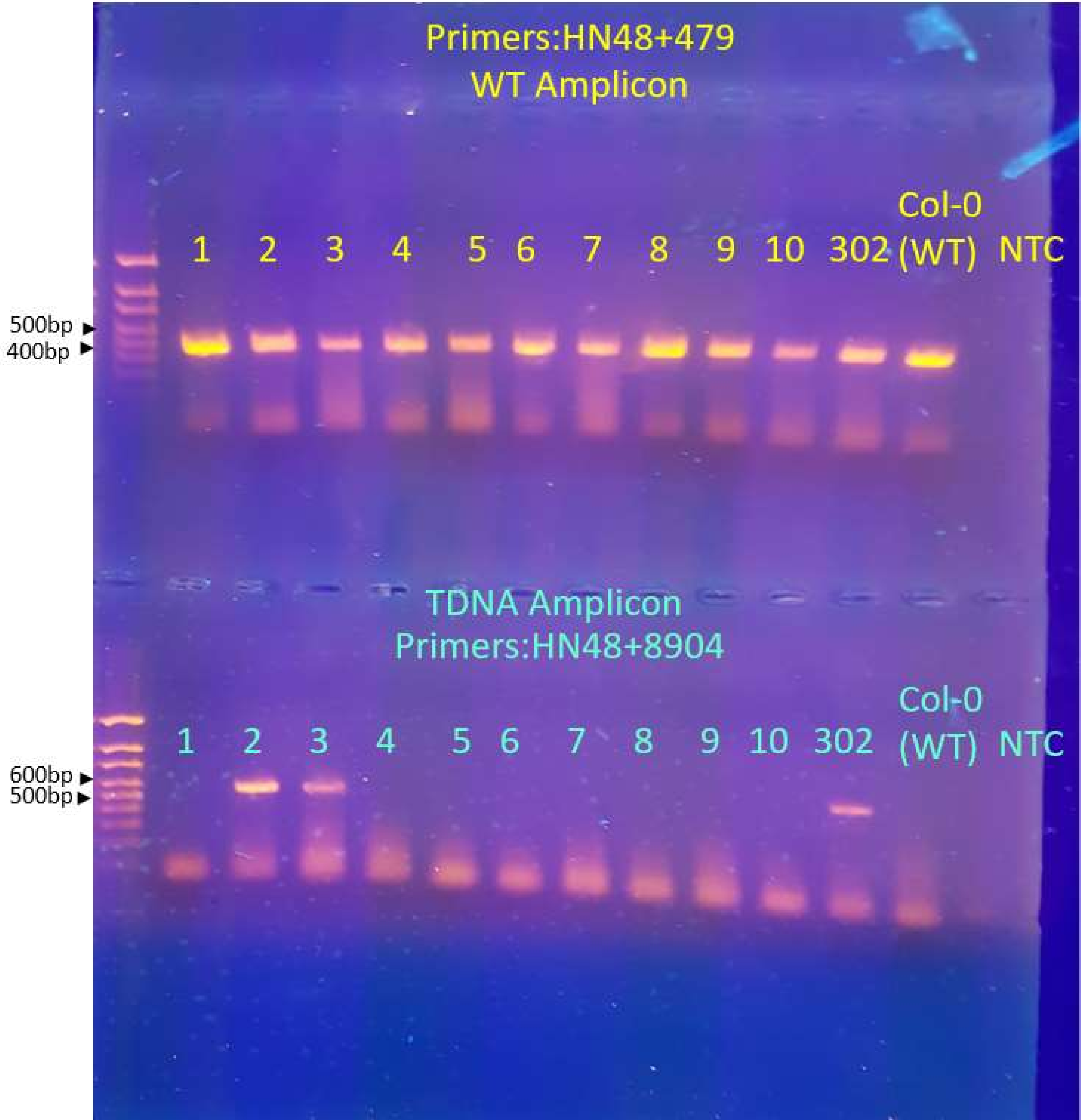

Figure S4: Gel images of GABI-Kat confirmation strategy
for presence of T-DNA insertion in RBL7 gene in 10 progeny of self-crossed heterozygous Arabidopsis
thaliana CS717302. Numbers 1–10 indicate individual progeny of the self-cross.

Arabidopsis ABRC lines	300	301	302	303	304	305	306	307	Col-0 (WT)
RBL7 Genotype	WT	WT	WT<br>TDNA	WT	WT<br>TDNA	WT<br>TDNA	WT<br>TDNA	WT<br>TDNA	WT

Table S1: Summary of the GABI-Kat confirmation strategy and the genotype of the RBL7 gene for the Arabidopsis thaliana lines from ABRC (CS717300-307).

Arabidopsis ABRC lines	302-1	302-2	302-3	302-4	302-5	302-6	302-7	302-8	302-9	302-10	302 (hetero)	Col-0 (WT)
RBL7 Genotype	WT	WT<br>TDNA	WT<br>TDNA	WT	WT	WT	WT	WT	WT	WT	WT<br>TDNA	WT

Table S2: Summary of the GABI-Kat confirmation strategy and the genotype of the RBL7 gene for 10 progeny of self-crossed heterozygous Arabidopsis thaliana CS717302 Numbers 1–10 indicate individual progeny of the self-cross.
